# Strengthening long-lasting insecticidal nets effectiveness monitoring using retrospective analysis of cross-sectional, population-based surveys across sub-Saharan Africa

**DOI:** 10.1038/s41598-018-35353-z

**Published:** 2018-11-20

**Authors:** Mark M. Janko, Thomas S. Churcher, Michael E. Emch, Steven R. Meshnick

**Affiliations:** 1 Duke Global Health Institute, Duke University, Durham, NC USA; 2 Department of Geography, University of North Carolina-Chapel Hill, Chapel Hill, NC USA; 3 Department of Biostatistics, University of North Carolina-Chapel Hill, Chapel Hill, NC USA; 4 Carolina Population Center, University of North Carolina-Chapel Hill, Chapel Hill, NC USA; 5 Faculty of Medicine, School of Public Health, Imperial College London, London, UK; 6 Department of Epidemiology, University of North Carolina-Chapel Hill, Chapel Hill, NC USA

## Abstract

Bed nets averted 68% of malaria cases in Africa between 2000 and 2015. However, concerns over insecticide resistance, bed net durability and the effectiveness of long-lasting insecticidal nets (LLIN) are growing. To assess the effectiveness of LLINs of different ages and insecticides against malaria, we conducted a population-based, cross-sectional study using data from 162,963 children younger than 5 years of age participating in 33 Demographic and Health and Malaria Indicator Surveys conducted in 21 countries between 2009 and 2016. We used Bayesian logistic regression to estimate associations between LLIN age, insecticide type, and malaria. Children sleeping under LLINs the previous night experienced 21% lower odds of malaria infection than children who did not (odds ratio [OR] 0.79; 95% Uncertainty Interval [UI] 0.76–0.82). Nets less than one year of age exhibited the strongest protective effect (OR 0.75; 95% UI 0.72–0.79), and protection weakened as net age increased. LLINs containing different insecticides exhibited similar protection (OR_deltamethrin_ 0.78 [0.75–0.82]; OR_permethrin_ 0.79 [0.75–0.83]; OR_alphacypermethrin_ 0.85 [0.76–0.94]). Freely-available, population-based surveys can enhance and guide current entomological monitoring amid concerns of insecticide resistance and bed net durability, and be used with locally-collected data to support decisions on LLIN redistribution campaign timing which insecticide to use.

## Introduction

Investment in malaria control has greatly reduced transmission. An estimated 663 million cases have been averted worldwide between 2000 and 2015, with 68% of them attributed to insecticide-treated nets^1^. Long-lasting insecticidal nets (LLINs) protect against malaria by acting as a physical barrier between mosquitos and humans, and by the insecticide repelling or killing susceptible mosquitoes^2–4^. The insecticide enhances public health impact by reducing mosquito density and helping maintain the net’s effectiveness after holes develop^2^.

Resistance to pyrethroids, the only class of insecticides approved for use in bed nets, has been reported across sub-Saharan Africa, though its public health significance remains unclear, with different studies giving contradictory results^2,5–16^. There is also emerging concern regarding the durability of LLINs, with some evidence suggesting that the serviceable life of a net is two, rather than three years^17,18^. This may be exacerbated by pyrethroid resistance, which has shortened the insecticidal impact of LLINs in experimental hut studies^19^. Many types of LLINs using several different pyrethroids are on the market. All LLINs recommended by the WHO have been evaluated in Phase II experimental hut trials^20^. Nevertheless, product quality may vary under field conditions. Some mechanisms of resistance are thought to confer protection against multiple insecticides^21^. Bioassay data indicates some degree of cross resistance within the pyrethroid class, though it is unclear whether this simple assay represents the more complex situation in the field^22^.

There is strong evidence for LLIN effectiveness, with a meta-analysis of nearly two dozen randomized controlled trials finding that insecticide-treated bed nets reduce parasite prevalence by 13%^3^. However, the operational effectiveness of LLINs in field conditions remains uncertain, especially given the number of different active ingredients used in LLINs and the different levels of insecticide resistance across sub-Saharan Africa. A recent study using population-based, cross-sectional surveys from seven countries across sub-Saharan Africa found that children sleeping under an insecticide-treated bed net the previous night experienced 24% lower odds of malaria^23^. This study, however, did not consider how effects might vary according to the insecticide nets were impregnated with, nor did it consider the age of the net. Conversely, a large prospective study conducted across four sub-Saharan African countries found that LLINs remained effective across a range of insecticide resistance levels^24^. However, combined, these two studies cover just eleven of 46 sub-Saharan African countries, a region that makes up 90% of the world’s malaria burden^25^. Therefore, information on bed nets impregnated with different insecticides, of different ages, and from more countries is needed.

Currently, the WHO recommends using either prospective, longitudinal studies or retrospective, population-based cross-sectional surveys to provide evidence for the durability and insecticide activity of nets under field conditions^26^. Prospectively monitoring malaria incidence against different interventions to determine their effectiveness is rare, however, owing to the considerable resources needed to conduct a longitudinal study. Conversely, population-based cross-sectional surveys have the advantage that they are often conducted at scale for other surveillance purposes, eliminating the extensive resources required for a prospective study. To our knowledge, however, population-based Demographic and Health Surveys (DHS) and Malaria Indicator Surveys (MIS) have yet to be used to investigate the effectiveness of LLINs of different ages and treated with different insecticides. Therefore, the aim of this study is to use DHS and MIS surveys to assess the effect of LLINs on the odds of malaria infection in children less than 5 years of age across 21 countries in sub-Saharan Africa. It investigates whether protection varies with LLINs of different ages and impregnated with different insecticides, and explores whether these effects vary by country.

## Results

A total 169,013 children younger than 5 years of age from 33 DHS and MIS surveys conducted in 21 countries from 2009 to 2016 across sub-Saharan Africa were initially screened for inclusion. A total of 886 children who did not have a malaria rapid diagnostic test (RDT), and 5,038 who slept under an untreated net were excluded. An additional 126 children were excluded due to missing data, yielding a final sample of 162,963 for analysis. Supplementary Figure 1 shows a flow diagram for the study.

Overall, 43,397 (27%) children tested positive for malaria by RDT, while 87,421 children (54%) were reported to have slept under an LLIN the previous night. Of those children sleeping under an LLIN, 42,018 (48%) slept under a net less than 1 year of age, followed by 22,853 (26%) who slept under a net 1–2 years of age. A total of 20,875 (24%) slept under a net over 2 years of age. The most common insecticide in use was deltamethrin, with 45,118 children (52%) sleeping under these nets, followed by permethrin (30,987 [35%]) and alphacypermethrin (4,892 [6%]). We were unable to identify the insecticide in 6,424 (7%) LLINs. Table 1 shows descriptive statistics for the entire sample, including potential confounders. Supplementary Table 1 shows these statistics for each survey, while Supplementary Table 2 shows the bed net brands identified and the insecticides used by those brands.

**Table 1:** Descriptive statistics for variables included in models. Total number of individuals in each group denoted (n) and standard deviation (sd) given where appropriate.

Variable	All surveys n (% of sample)
**Malaria**	
RDT+	43,397 (26.6)
RDT**−**	119,566 (73.4)
Age in years – mean (sd)	2.8 (1.4)
**Sex**	
Boys	82,203 (50.4)
Girls	80,760 (49.6)
**LLIN Use**	
Slept under LLIN	87,421 (53.6)
Did not sleep under LLIN	75,542 (46.4)
**Net Age**	
<1 year	42,018 (25.8)
1–2 years	22,853 (14.0)
2–3 years	9,243 (5.7)
>3 years	11,632 (7.1)
Unknown age	1,675 (1.0)
**Insecticide**	
Deltamethrin	45,118 (27.7)
Permethrin	30,987 (19.0)
Alphacypermethrin	4,892 (3.0)
Unknown insecticide	6,424 (3.9)
**Housing quality**	
Natural	28,533 (17.5)
Mixed natural/rudimentary	25,322 (15.5)
Mixed natural/finished	26,310 (16.1)
Rudimentary	4,456 (2.7)
Mixed rudimentary/finished	19,330 (11.9)
Finished	56,870 (34.9)
Other	2,142 (1.3)
**Community type**	
Urban	41,791 (25.6)
Rural	121,172 (74.4)

Bayesian logistic regression models assessing the effect of any LLIN, LLIN age, and LLIN insecticide had the same predictive performance, while the model for LLIN age by insecticide interaction had the worst predictive performance. Similarly, the model for any LLIN was the most parsimonious based on DIC, while the models for LLIN age and LLIN insecticide had similar DICs. Supplementary Table 3 shows measures of fit for the models considered.

**Figure 1:**
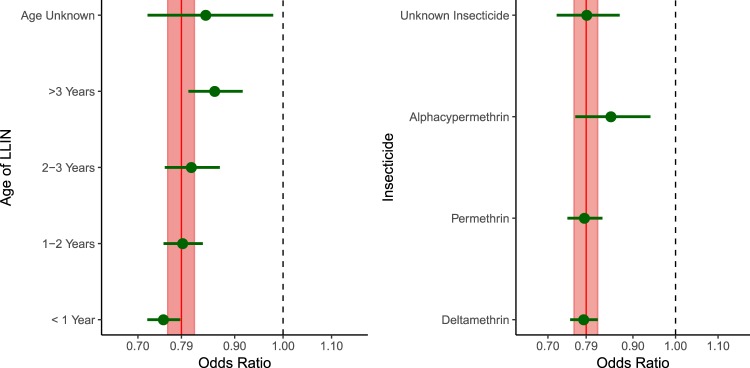
Effects of sleeping under an LLIN by age (left) and by insecticide (right). Note: The solid red vertical line represents the effect of sleeping under any LLIN (regardless of age and insecticide), while the shaded region represents the 95% uncertainty interval around this estimate. The green points represent the point estimates of sleeping under a net of a given age (left) or insecticide (right), while the horizontal green lines represent 95% uncertainty intervals. The dashed vertical line at 1 shows the null value of no difference between users of bed nets of a given age (left) or impregnated with a given insecticide (right) and children who did not sleep under a bed net of any kind.

Across all surveys, children sleeping under an LLIN had 21% lower odds of malaria infection (OR 0.79; 95% UI 0.76–0.82, Fig. 1, Supplementary Table 4). This effect varied slightly by the age of the net, with nets less than 1 year of age having the strongest protective effects (OR 0.75; 95% UI 0.72–0.79), followed by nets 1–2 years of age (OR 0.79; 95% UI 0.75–0.83), nets 2–3 years of age (OR 0.81; 95% UI 0.76–0.87), and nets over 3 years of age (OR 0.86; 95% UI 0.80–0.92). There was less variability by insecticide across all surveys, with deltamethrin (OR 0.78; 95% UI 0.75–0.82), permethrin (OR 0.79; 95% UI 0.75–0.83), and nets with an unknown insecticide (OR 0.79; 95% UI 0.72–0.87) yielding similar effects to the overall average. Alphacypermethrin-impregnated nets exhibited a slightly weaker protective effect, with children under these nets having 15% lower odds of infection than children not sleeping under any net (OR 0.85; 95% UI 0.76–0.94).

**Figure 2:**
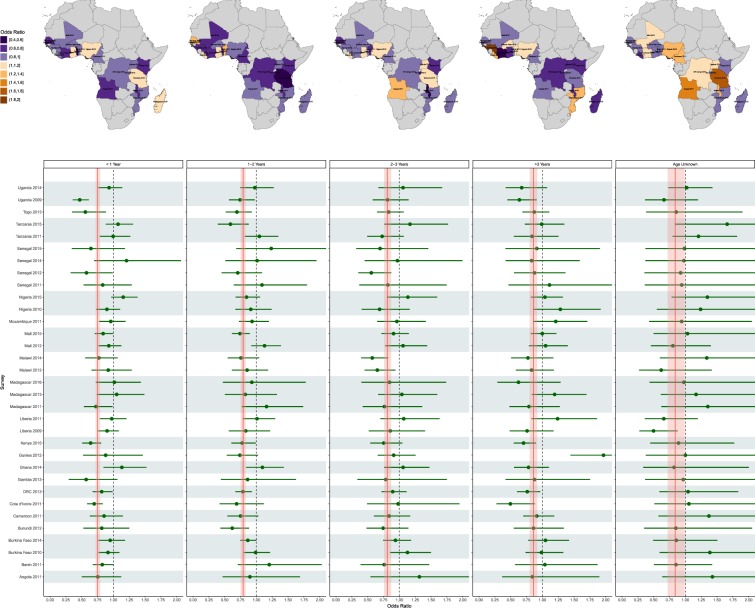
Effects of sleeping under an LLIN of different ages. Top panel: Estimates of the effect of sleeping under a LLIN of a given age using each country’s most recent survey. Bottom panel: Forest plot showing the same estimates for all surveys. The solid red vertical line represents the average effect of sleeping under an LLIN of the specifided age across all surveys combined, while the shaded region represents the 95% uncertainty interval around this estimate. The horizontal shaded regions group surveys by country. The dashed vertical line at 1 shows the null value of no difference between users who slept under an LLIN of a given age and children who did not sleep under a net of any kind.

The odds of being diagnosed malaria positive using nets of different ages appears to vary slightly across surveys (Fig. 2, Supplementary Table 5). For example, in Togo, which had a prevalence of 40% in 2013, children sleeping under a net less than 1 year of age experienced 44% lower odds of infection than children not sleeping under any net (OR 0.56, 95% UI 0.35–0.88). This protection deteriorated as the age of nets increased, with children sleeping under nets 1–2 years of age experiencing 31% lower odds of infection (OR 0.69 95% UI 0.52–0.93), followed by nets 2–3 years of age (OR 0.83, 95% UI 0.65–1.07), with nets over 3 years of age conferring little or no protection (OR 0.87, 95% UI 0.68–1.11). Broadly speaking, the impact of net age is consistent across most surveys and LLINs of all ages are predicted to have a protective effect.

**Figure 3:**
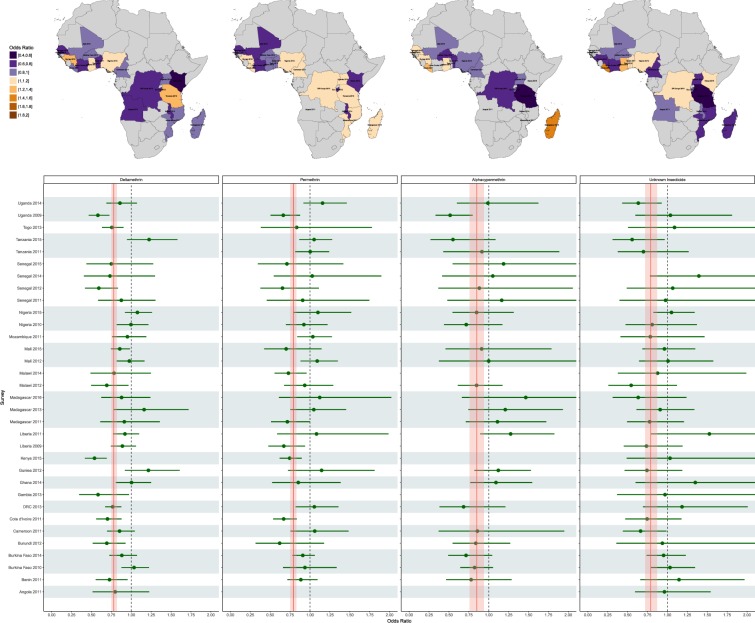
Effects of sleeping under an LLIN treated with a given insecticide. Top panel: Estimates of the effect of sleeping under a LLIN impregnated with a given insecticide using each country’s most recent survey. Bottom panel: Forest plot showing the same estimates for all surveys. The solid red vertical line represents the average effect of sleeping under an LLIN impregnated with the specified insecticide across all surveys combined, while the shaded region represents the 95% uncertainty interval around this overall estimate. The gray horizontal shaded regions group surveys by country. The dashed vertical line shows the null value of 1, indicating there is no difference between users of nets of a given insecticide and children who did not use a net of any kind. Points were omitted when LLINs with that insecticide were not identified in the specific survey for that country.

The effect of using LLINs with different insecticides also appears to vary across surveys (Fig. 3, Supplementary Table 6). In some surveys, certain insecticides appear to confer less direct protection than others whilst in other areas there appears to be no difference. For example, in Benin in 2011, where prevalence was 27%, deltamethrin-based nets had a direct protective effect (OR 0.72, 95% UI 0.55–0.95), while permethrin-based nets appear to provide little or no direct benefit (OR 0.89, 95% UI 0.72–1.10). Conversely, in Tanzania in 2015, where prevalence was 12%, and where alphacypermethrin-based nets were associated with lower odds of infection, permethrin and deltamethrin nets appear to confer no protection. Finally, in Uganda, the protective effects of all three insecticides appear to have disappeared between the 2009 and 2014.

## Discussion

This analysis assesses the direct personal protection an LLIN provides, and reconfirms that LLINs are hugely effective in reducing the prevalence of malaria, with findings similar to other multicountry studies^1,23^. The full effect is likely greater, since insecticides on the net kill mosquitoes, protecting users and non-users alike. Assessing this community benefit is prohibitively expensive for the purposes of routine monitoring as it requires costly community randomized control trials^3^. Comparison of malaria in LLIN users and non-users is increasingly common so it is important to understand how results should be interpreted^24^.

This work shows that LLINs of all ages provide direct personal protection. Younger nets appear to provide more protection than older nets. This result appears intuitive as LLINs physically degrade over time. LLINs also lose bio-efficacy as they age, making it more likely that a mosquito will enter a holed net. Whether the observed reduction in protection is due to physical degradation, loss of bio-efficacy, or both cannot be determined here. Nevertheless, the loss of protection each year has implications for the regularity of LLIN distribution campaigns. The best time to repeat distribution campaigns is not clear. LLIN durability studies and the use of old nets in experimental hut trials can provide entomological measures of LLIN efficacy. The small number and focality of these studies make generalizing to a country’s wider population difficult and do not show the direct effectiveness of the intervention. This study overcomes these issues and provides a population-wide, real-world estimate of the serviceable life of LLINs. Ultimately, the decision will depend on how LLIN coverage declines over time and the economics of providing new nets against alternative interventions such as indoor residual spraying (IRS).

Overall there is little difference in the personal protection provided by LLINs with different types of insecticide. This is consistent with recent work which suggests there is strong cross resistance between the different insecticides within the pyrethroid class^22^. There is considerable variation between surveys and National Malaria Control Programmes may wish to use country specific results to justify more thorough entomological work. Care should be taken, as LLINs of different types might not be evenly distributed across the country and the background malaria prevalence might vary by LLIN type. While 48% of survey clusters reported LLINs with different active ingredients, small cluster sizes precluded a thorough direct comparison of the insecticidal action of the nets within clusters (median cluster size: 12 children, IQR 7–20). There is some uncertainty about how user versus non-user data should be used to investigate the effectiveness of insecticides in LLINs. Sleeping under a structurally sound LLIN will always provide users with some direct personal protection. This has been illustrated using transmission dynamics mathematical models of malaria parameterized with experimental hut trials conducted in areas with different levels of pyrethroid resistance^19^. A difference in the odds of malaria without any other information is insufficient evidence to support definitive statements on the effectiveness of the insecticides in LLINs. Failing to find a difference between users and non-users (as is sometimes the case) may be due to LLIN use in an area being correlated with mosquito activity, with highly bitten people more likely to use a net^24^. The analysis presented here overcomes this issue by grouping children together in relatively small geographic clusters where malaria exposure is likely to be more consistent. Nevertheless, the focality of the disease means that this effect can never be excluded completely.

One of the rationale for including insecticide on bed nets was that it would deter and kill mosquitoes trying to enter a net as holes appear. It follows that the insecticidal component of the direct personal protection provided by LLINs will become more important as nets age and acquire more holes. Evidence suggests that the age at which a mosquito can overcome the action of the insecticide on a LLIN declines as the level of resistance in the mosquito population increases^19^. It therefore seems likely that an early sign of resistance might be the more rapid decline in personal protection provided by LLINs as they age. The statistical models presented here gave a less parsimonious fit to these data when an interaction term between insecticide type and LLIN age was included. This would suggest either that overall there was no impact of insecticide resistance, or that the impact was consistent across LLIN insecticides. Given that, on average, the level of pyrethroid resistance was relatively low across Africa at the time the surveys were conducted^27,28^, and that there seems to be substantial cross-resistance between insecticides within the pyrethroid class^22^, further work is needed to understand how these analyses can be used to investigate the public health impact of pyrethroid resistance.

The statistical analyses of large-scale, routinely collected data has a number of strengths. First, DHS and MIS surveys provide detailed data on malaria status, LLIN use, and LLIN characteristics, together with important demographic and socioeconomic confounders, for one of the populations at highest risk of malaria—children under 5 years of age. Second, the data are free and publicly available, such that researchers or government agencies can readily use them to monitor components of LLIN effectiveness over time as additional surveys are conducted. Third, the surveys allow effectiveness measurement against malaria directly, a more relevant outcome than vector mortality measured in entomological surveillance. Finally, the surveys are representative regionally within countries as well, facilitating a more detailed understanding within a given country.

Our study has several limitations. First, IRS is an important confounder that we were unable to address. Whether or not a household was sprayed is a standard question in the surveys, but ministries of health select which questions to ask, and this question was only asked in some surveys. However, the insecticide used is not reported and the persistence of different sprays varies^29^. Second, LLIN use, age and brand was assessed through questionnaires and are subject to reporting and misclassification bias. Another potential explanation is that older nets were re-treated, which we did not investigate. Whether or not an LLIN was re-treated (and when) is a standard question in the malaria module of the DHS and MIS, but it was only asked in some surveys. Finally, we cannot ignore residual confounding. Seasonal rainfall and other environmental variables represent potential factors which were not fully accounted for in the analysis. Addressing this confounding necessarily involves understanding more about the characteristics and primary species in the vector population, which may respond to environmental exposures and interact with LLINs in different ways. For example, recent work from the DR Congo found that increased rainfall affected biting rates in *An. gambiae* and *An. funestus* in different ways. Similarly, the vector population in that study varied over time and space^30^. In light of this variability, and because there was no entomological monitoring in the DHS/MIS survey data, we cannot adequately account for how seasonal variability or environmental exposures such as rainfall may confound our results. Further, we cannot meaningfully account for how changes in vector composition or behavior may impact our estimates. For example, there is evidence that biting behavior may shift outdoors in response to large scale-ups of LLIN distributions, which could result in null estimates of LLIN effectiveness independent of age or insecticide^31^.

To our knowledge, this is the first study to use population-based DHS and MIS survey data to assess the effect of LLINs of different ages and insecticides on malaria transmission. This approach builds on the previous multi-country study by Lim *et al*., which used DHS and MIS surveys to assess the protective benefits of LLIN ownership and use on malaria parasitemia and child mortality^23^. While the study by Lim *et al*. points to a low cost way that LLIN performance can be assessed in field conditions, our work highlights how these data can further be used to supplement current entomological surveillance activities within a country, and to facilitate decision-making around what nets to deploy, where, and when. Importantly, the need for this type of routine monitoring is becoming more acute as increasing number of LLIN brands enter the market. These LLINs vary substantially in the dose of insecticide used, manufacture (for example whether the insecticide is coated on the net or incorporated) and net dernier, amongst others. This is especially the case for LLINs with the synergist piperonyl butoxide (PBO), a synergist which can increase pyrethroid efficacy, that have recently been recommend for use by the WHO in areas of pyrethroid resistance^32^. These LLINs contain substantially different quantities of PBO. Additionally, a recent cluster-randomized controlled trial comparing nets impregnated with permethrin and pyriproxyfen (a mosquito growth inhibitor) to permethrin-only nets found that the former reduced clinical incidence of malaria by 12%, suggesting that these nets may also enter the market in the future^33^. There is also growing interest in the possible reintroduction of untreated nets in the context of insecticide resistance. Investigating the effects of untreated nets was beyond the scope of our work here. Regardless, as LLIN distributions continue, and as new classes of nets emerge into the market, maintaining their efficacy will be of paramount importance. DHS and MIS surveys, which are conducted regularly within countries, can facilitate this effort.

## Methods

### Study design and data sources

This study uses cross-sectional data. We used STROBE guidelines to guide reporting, and include a checklist in the supplementary material.

We obtained data on malaria and LLIN use in children younger than 5 years of age from publicly available DHSs and MISs conducted between 2009 and 2016. Briefly, DHSs are two-stage cluster household surveys designed to provide nationally and sub-nationally representative estimates across a number of domain areas among children less than 5 years of age, and women and men of reproductive age. MISs follow the same survey design but are more limited in scope than a DHS. All surveys collect extensive demographic and socioeconomic data from participants. Further details for these surveys are available elsewhere^34–36^. Since 2007, the WHO has recommended that all nets in use be LLINs, since they are designed to main insecticidal activity for three years in field conditions^26,37–39^. We restrict this study to only consider surveys from 2009 onwards to help ensure that the net type is consistent. Children were eligible to be included if they were less than 5 years of age, were tested for malaria by RDT, and had no missing data on exposure, outcome, or confounding variables.

### Outcome and exposure measures

The primary outcome in this study is the RDT-based malaria status of each child. Children were tested for malaria using both RDT and microscopy, and we chose to use the RDT-based results because RDT-based testing is more widely used, accounting for 71% of malaria diagnostic testing^40^. The exposure measures of interest are (1) whether or not a child slept under an LLIN the previous night, (2) whether that net was less than 1 year of age, 1–2 years of age, 2–3 years of age, over 3 years of age, or of unknown age and (3) whether or not the net was impregnated with deltamethrin, permethrin, alphacypermethrin, or other insecticide. We determined the age of the net based on how many months ago survey respondents reported receiving it, and identified the insecticide used using the reported brand of net in the survey.

### Potential confounders

Potential confounders included in this analysis are: age in years, sex, urban/rural status, and housing quality, which was based on roof and wall construction materials, and coded as natural (e.g. no walls/roof or cane/palm/trunks for walls, thatch/palm leaf for roof), rudimentary (e.g. wood with mud for walls, wood planks for roof), or finished (e.g. cement/tin for walls, metal or shingles for roof). These categories represent measures of both socioeconomic status, as well as different barriers to mosquito entry, with natural materials providing the greatest access, and finished materials the greatest barrier. We categorized homes as constructed from natural, mixed natural/rudimentary, mixed natural/finished, rudimentary, mixed rudimentary/finished, or finished materials.

### Statistical methods

We used multilevel Bayesian logistic regression to estimate associations between a child’s malaria status and their LLIN use. We fit four models to all surveys combined. The first model estimated the effect of sleeping under an LLIN of any type. The second model estimated the effects of LLINs of different ages. The third model estimated the effects of different insecticides. The fourth model estimated LLIN age and insecticide interactions. We initially withheld 50% of the data for fitting and compared out-of-sample predictive performance on the remaining 50% of the data using Brier scores^41^. We then refit the models to the full data set and compared fit using the Deviance Information Criterion (DIC)^42^. We further stratified by survey to explore how the effects vary by country. All models controlled for potential confounders and included an intercept that varied independently between survey clusters to account for the survey design. For models using all surveys combined, regression coefficients were assigned weakly informative standard normal prior distributions. For stratified models, we assigned weakly informative normal prior distributions with mean zero and standard deviation one half, corresponding to 95% prior belief that all odds ratios are between 0.37 and 2.66. Data management and modeling were done using R statistical software (version 3.3.1). Model fitting was done using integrated nested Laplace approximation (INLA)^43^. Additional model details are contained in the supplementary material.

## Electronic supplementary material

Supplementary Material

## Data Availability

The data for this work are freely available from the DHS Program at www.dhsprogram.com.
