# Supplementary material for: Strengthening long-lasting insecticidal nets effectiveness monitoring using retrospective analysis of cross-sectional, population-based surveys across sub-Saharan Africa
Source: Sci Rep. 2018 Nov 20;8:17110. doi: 10.1038/s41598-018-35353-z (PMC6244007; doi:10.1038/s41598-018-35353-z)
Supplement: Supplementary file 1 — Supplementary Material [file 41598_2018_35353_MOESM1_ESM.pdf]

Supplementary Material for: Strengthening long-lasting insecticidal nets effectiveness monitoring using retrospective analysis of cross-sectional, population-based surveys across sub-Saharan Africa.

Mark M. Janko[a,b,c,d*], Thomas S. Churcher[e], Michael E. Emch[b,d,f], Steven R. Meshnick[f]

a. Duke Global Health Institute, Duke University, Durham, NC, USA

b. Department of Geography, University of North Carolina-Chapel Hill, Chapel Hill, NC, USA

c. Department of Biostatistics, University of North Carolina-Chapel Hill, Chapel Hill, NC, USA

d. Carolina Population Center, University of North Carolina-Chapel Hill, Chapel Hill, NC, USA

e. Faculty of Medicine, School of Public Health, Imperial College London, London, UK

f. Department of Epidemiology, University of North Carolina-Chapel Hill, Chapel Hill, NC, USA

* corresponding author

Email: mark.janko@duke.edu

STROBE Checklist

	Item No	Recommendation
**Title and abstract**	1	(*a*) Indicate the study's design with a commonly used term in the title or the abstract<br><br>**We state that this is a population-based, cross-sectional study in the title.**
		(*b*) Provide in the abstract an informative and balanced summary of what was done and what was found<br><br>**We describe the study populations, outcome measures, exposure measures, statistical methods, and results of our analysis in the abstract.**
**Introduction**		
Background/rationale	2	Explain the scientific background and rationale for the investigation being reported<br><br>**Scientific Background: We provide a comprehensive summary of previous work on the relationship between insecticide resistance and malaria, including a number of trials. We note that these studies tend to be conducted in a small number of settings, limiting generalizability. Given that bed net decisions are often made at the country level, we argue that more work at the population level is needed.**<br><br>**Rationale: We write in the introduction that insecticide resistance is expanding across Africa, and that there is growing concern regarding the durability of bed nets in field conditions.**
Objectives	3	State specific objectives, including any prespecified hypotheses<br><br>**We state that our objective is to "examine the relationship between agriculture, the mosquito population, and malaria risk using data from a population-based cross-sectional survey of children under 5 years of age living in the Democratic Republic of Congo…and contemporaneous entomological monitoring data collected over time across DRC's ecological zones."**

		**We state that our hypothesis is that increasing exposure to agriculture is associated with increased malaria risk, and seek to understand how changes in vector behaviour may be a mechanism underlying this hypothesized increase.**
**Methods**		
Study design	4	Present key elements of study design early in the paper<br><br>**We state both in the title and in the introduction that this is a cross-sectional study. We further describe the study populations (children under 5 years of age) in detail, including sample sizes and selection criteria in the methods section.**
Setting	5	Describe the setting, locations, and relevant dates, including periods of recruitment, exposure, follow-up, and data collection<br><br>**We describe in detail the country settings, the year each survey was conducted, and include a table (Table 1) with summary measures for the outcome and exposure measures of interest for each survey.**
Participants	6	(*a*) Give the eligibility criteria, and the sources and methods of selection of participants<br><br>**We describe the eligibility criteria in detail in the methods section. Briefly, they are children under 5 years of age living who were tested for malaria by RDT, either slept under an LLIN or did not sleep under any net, and had no missing covariate information. Only 126 individuals had missing data.**
Variables	7	Clearly define all outcomes, exposures, predictors, potential confounders, and effect modifiers. Give diagnostic criteria, if applicable<br><br>**We define outcomes and exposures in the description of the study population, and dedicate a separate section to confounding variables and how they were measured.**

Data sources/ measurement	8*	For each variable of interest, give sources of data and details of methods of assessment (measurement). Describe comparability of assessment methods if there is more than one group<br><br>**We describe the source of each variable (outcome, exposure, or confounder).**
Bias	9	Describe any efforts to address potential sources of bias<br><br>**We address sources of bias in previous work, how our work helps to address such biases, and further discuss possible bias in our work. For example, we note that our measures of the exposures are subject to misclassification bias and/or reporting bias.**
Study size	10	Explain how the study size was arrived at<br><br>**We provide a description of the selection criteria and provide a study flow diagram as Supplementary Figure 1 in our study.**
Quantitative variables	11	Explain how quantitative variables were handled in the analyses. If applicable, describe which groupings were chosen and why<br><br>**We provide a detailed description of how quantitative variables were handled (e.g. centering and scaling). We also describe that groupings were chosen based on how the data were collected (e.g. with regard to the housing characteristics variables).**
Statistical methods	12	(*a*) Describe all statistical methods, including those used to control for confounding<br><br>**We describe our statistical methods used in the main text of the paper, together with a discussion of weakly informative prior distributions and how we used them to have the model yield parameter estimates within an epidemiologically relevant range.**
		(*b*) Describe any methods used to examine subgroups and interactions<br><br>**We stratified by survey and considered the effects of nets by age and by insecticide separately, as well as their interaction**

		(*c*) Explain how missing data were addressed<br><br>**We state that this is a complete case analysis, as there were only 126 study subjects out of 169 013 with any missing data.**
		(*d*) If applicable, describe analytical methods taking account of sampling strategy<br><br>**In our description of the methodological approach, we note that we specify a multilevel model to account for the sampling strategy of the survey. Our model has the following general form:**<br><br>$$y_{ij} = \boldsymbol{x}_{ij}^T\boldsymbol{\beta} + \theta_j$$<br><br>**where y_{ij} is the observed malaria outcome for child $\boldsymbol{i}$ in survey cluster $\boldsymbol{j}$, $\boldsymbol{x}_{ij}^T$ is a $\mathbf{1} \times \boldsymbol{p}$ row vector of covariates for child $\boldsymbol{i}$ in cluster $\boldsymbol{j}$, $\boldsymbol{\beta}$ is a $\boldsymbol{p} \times \mathbf{1}$ vector of regression coefficients linking the covariates to the response (through a logit link), while θ_j represents a unique cluster-level random effect.**
		(*e*) Describe any sensitivity analyses<br><br>**We implement 4 different multilevel Bayesian models to investigate whether or not different bed net exposures yield better fits to the data. We further describe how we assess model fit and provide fit statistics in Supplementary Table 2.**
**Results**		
Participants	13*	(a) Report numbers of individuals at each stage of study—eg numbers potentially eligible, examined for eligibility, confirmed eligible, included in the study, completing follow-up, and analysed<br><br>**This information is included in the methods section, as well as in supplementary figure 1.**
		(b) Give reasons for non-participation at each stage<br><br>**The mothers for all eligible participants assented to their children being included in the study.**

		(c) Consider use of a flow diagram<br><br>**We include a flow diagram in supplementary figure 1.**
Descriptive data	14*	(a) Give characteristics of study participants (eg demographic, clinical, social) and information on exposures and potential confounders<br><br>**We include this information in Tables 1 and 2**
		(b) Indicate number of participants with missing data for each variable of interest<br><br>**A total of 126 individuals had missing data.**
Outcome data	15*	Report numbers of outcome events or summary measures<br><br>**We begin the results section by summarizing the outcome and exposure measures for the entire sample, and further provide these summary measures by survey, since we also stratify our analysis by survey.**
Main results	16	(*a*) Give unadjusted estimates and, if applicable, confounder-adjusted estimates and their precision (eg, 95% confidence interval). Make clear which confounders were adjusted for and why they were included<br><br>**We do not include a discussion of unadjusted estimates owing to space limitations. Additionally, our literature review indicated that confounding is an important limitation of studies on the insecticide-malaria relationship, and we therefore focus on addressing this confounding by including confounders that are otherwise unavailable in other studies.**
		(*b*) Report category boundaries when continuous variables were categorized<br><br>**We did not categorize continuous variables.**

		(*c*) If relevant, consider translating estimates of relative risk into absolute risk for a meaningful time period<br><br>**Not applicable. We report Odds Ratios.**
Other analyses	17	Report other analyses done—eg analyses of subgroups and interactions, and sensitivity analyses<br><br>**We report results for the 3 models that yielded similar fit. We present the results from the 4th model in the supplementary appendix, and report the main results (i.e. those from the best-fitting model) in the main text.**
**Discussion**		
Key results	18	Summarise key results with reference to study objectives<br><br>**We provide a broad summary that our findings suggest that bed nets treated with different insecticides and of different ages are effective across Africa, but that there is variability across countries.**
Limitations	19	Discuss limitations of the study, taking into account sources of potential bias or imprecision. Discuss both direction and magnitude of any potential bias<br><br>**We discuss potential bias in our discussion. Specifically, we discuss that misclassification and reporting bias may undermine our results. Further, we note that we cannot draw inferences on the adult population, since adults are not tested for malaria.**
Interpretation	20	Give a cautious overall interpretation of results considering objectives, limitations, multiplicity of analyses, results from similar studies, and other relevant evidence<br><br>**We work to ensure a cautious interpretation by using cautious language (i.e. the words "suggest" and "may" and "appear"), e.g.:**<br><br>**-"…our analysis based on 2011 survey data suggested that nets treated with deltamethrin exhibited a weak protective effect…"**<br><br>**-" The effect of using nets of different ages appears to vary across surveys."**

		**- “permethrin-treated nets appear to provide little or no protective benefit (OR 0.89, 95% UI 0.72 – 1.10)”**<br><br>**“-Thus, the observed lack of effectiveness in a net treated with a given insecticide may not be due to resistance, but to IRS or other pesticide spraying that kills mosquitoes before they have the opportunity to make contact with a net.”**
Generalisability	21	Discuss the generalisability (external validity) of the study results<br><br>**We note that one of the strengths of this study is that it relies on population-based surveys of children under 5 years of age, suggesting that the results are generalizable to the population of children under 5 years of age.**
**Other information**		
Funding	22	Give the source of funding and the role of the funders for the present study and, if applicable, for the original study on which the present article is based<br><br>**We include the following statements in the manuscript:**<br><br>**The sponsors of the study had no role in study design, data collection, data analysis, data interpretation, or writing of the report. The corresponding author had full access to all the data in the study and had final responsibility for the decision to submit for publication. Parental consent for children’s participation in the DHS and MIS surveys was obtained by the DHS Program.**<br><br>**The authors acknowledge support from the National Institutes of Health (grant 5R01AI107949 to Steven R. Meshnick), the National Science Foundation (grant BCS-1339949 to Michael Emch). Mark Janko received support from the Royster Society of Fellows at UNC-CH. Mark Janko was supported by the Population Research Infrastructure Program awarded to the Carolina Population Center (P2C HD050924) by the *Eunice Kennedy Shriver* National Institute of Child Health and Development.**

Supplementary Figure 1. Study Flow Diagram

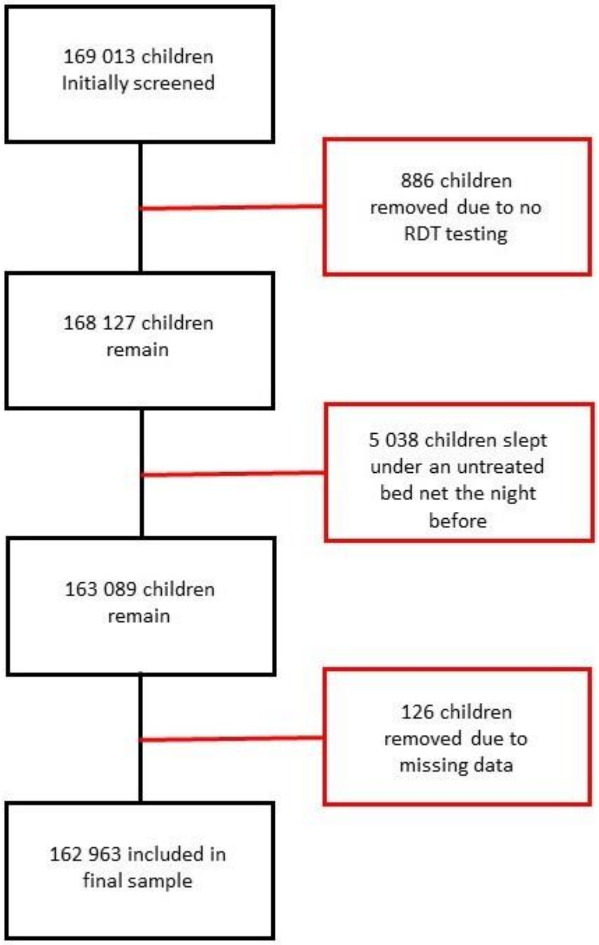

Supplementary Table 1. Bed net brands by insecticide

**Insecticide**	**Bed net brand**
Deltamethrin	Permanet; Net Protect; Tuzanet; Mamanet; Dawa Plus; Life Net; Yorkool; Serena; Icon Life; K-onet/K-ONET
Permethrin	Olyset
Alphacypermethrin	Duranet; Interceptor; Magnet; Royal Sentry; BASF
Other	Joia; Slavo; Safi net; NETTO; Sentinelle; Seguro; Tsaralay; Milay; Super Mosquitaire; Dawnet

Supplementary Table 2. Model fit statistics

Model fit for exposure of interest	Brier score	DIC
Child slept under an LLIN	0.24	148 109
Child slept under an LLIN of a given age	0.24	148 128
Child slept under an LLIN of a given insecticide	0.24	148 129
Child slept under an LLIN of a given age and insecticide	0.28	148 232

Notes: For both measures (brier score and DIC), lower values indicate better fit

Supplementary Table 3. Results from all models describing A) the effect of sleeping under any LLIN across all surveys; B) the effect of sleeping under an LLIN of different ages across all surveys; C) the effect of sleeping under an LLIN treated with different insecticides across all surveys; and D) the effect of sleeping under an LLIN of different ages and treated with different insecticides across all surveys.

Model	Variable	OR (95% UI)
**A. Any LLIN use**	**LLIN**	0.79 (0.76 – 0.82)
**B. LLIN use by age**	**LLIN age**	
	< 1 year	0.75 (0.72 – 0.79)
	1-2 years	0.79 (0.75 – 0.83)
	2-3 years	0.81 (0.76 – 0.87)
	>3 years	0.86 (0.80 – 0.92)
	Age unknown	0.84 (0.72 – 0.99)
**C. LLIN use by insecticide**	**LLIN insecticide**	
	Deltamethrin	0.78 (0.75 – 0.82)
	Permethrin	0.79 (0.75 – 0.83)
	Alphacypermethrin	0.85 (0.76 – 0.94)
	Unknown insecticide	0.79 (0.72 – 0.87)
**D. LLIN use by age and insecticide**	**LLIN insecticide x age**	
	Deltamethrin	
	< 1 year	0.91 (0.30 – 2.83)
	1-2 years	0.90 (0.29 – 2.78)
	2-3 years	1.07 (0.35 – 3.32)
	>3 years	0.99 (0.32 – 3.05)
	Age unknown	1.04 (0.33 – 3.29)
	Permethrin	
	< 1 year	0.89 (0.29 – 2.76)
	1-2 years	1.00 (0.32 – 3.09)
	2-3 years	0.95 (0.31 – 2.96)
	>3 years	0.94 (0.30 – 2.92)
	Age unknown	1.14 (0.36 – 3.58)
	Alphacypermethrin	
	< 1 year	1.00 (0.32 – 3.13)
	1-2 years	1.02 (0.33 – 3.19)
	2-3 years	1.01 (0.32 – 3.17)
	>3 years	1.17 (0.37 – 3.68)
	Age unknown	0.74 (0.21 – 2.60)
	Unknown insecticide	
	< 1 year	1.10 (0.35 – 3.42)
	1-2 years	1.01 (0.32 – 3.15)
	2-3 years	0.85 (0.27 – 2.68)
	>3 years	0.90 (0.29 – 2.81)
	Age unknown	0.99 (0.30 – 3.28)

**Supplementary Table 4. Stratified results showing the effects of sleeping under an LLIN of different ages by survey.**				
**Survey**	**LLIN age**	**Odds Ratio**	**Lower 95% UI**	**Upper 95% UI**
**Angola 2011**	< 1 Year	0.75	0.50	1.12
	1-2 Years	0.90	0.47	1.69
	2-3 Years	1.32	0.54	3.14
	>3 Years	0.84	0.37	1.90
	Age Unknown	1.43	0.64	3.19
**Benin 2011**	< 1 Year	0.82	0.67	1.01
	1-2 Years	1.20	0.71	2.03
	2-3 Years	0.76	0.39	1.47
	>3 Years	1.04	0.57	1.87
	Age Unknown	0.85	0.50	1.42
**Burkina Faso 2010**	< 1 Year	0.91	0.76	1.10
	1-2 Years	0.99	0.80	1.22
	2-3 Years	1.13	0.85	1.50
	>3 Years	0.99	0.73	1.33
	Age Unknown	1.39	0.59	3.29
**Burkina Faso 2014**	< 1 Year	0.95	0.76	1.18
	1-2 Years	0.86	0.75	1.00
	2-3 Years	0.94	0.74	1.19
	>3 Years	1.05	0.78	1.42
	Age Unknown	0.86	0.49	1.51
**Burundi 2012**	< 1 Year	0.82	0.53	1.25
	1-2 Years	0.62	0.43	0.88
	2-3 Years	0.75	0.48	1.14
	>3 Years	0.86	0.55	1.34
	Age Unknown	0.85	0.35	2.06
**Cameroon 2011**	< 1 Year	0.85	0.63	1.15
	1-2 Years	0.75	0.54	1.03
	2-3 Years	0.84	0.60	1.17
	>3 Years	0.91	0.70	1.19
	Age Unknown	1.37	0.58	3.28
**Cote d'Ivoire 2011**	< 1 Year	0.70	0.58	0.83
	1-2 Years	0.69	0.42	1.12
	2-3 Years	0.98	0.49	1.95
	>3 Years	0.50	0.28	0.88
	Age Unknown	1.06	0.51	2.19
	< 1 Year	0.82	0.67	0.99

**DR Congo 2013**	1-2 Years	0.79	0.67	0.93
	2-3 Years	0.90	0.72	1.11
	>3 Years	0.76	0.60	0.97
	Age Unknown	1.04	0.59	1.84
**Gambia 2013**	< 1 Year	0.57	0.29	1.06
	1-2 Years	0.86	0.44	1.62
	2-3 Years	0.78	0.34	1.75
	>3 Years	0.88	0.42	1.75
	Age Unknown	0.97	0.37	2.53
**Ghana 2014**	< 1 Year	1.13	0.84	1.52
	1-2 Years	1.10	0.84	1.43
	2-3 Years	1.06	0.76	1.47
	>3 Years	0.78	0.55	1.10
	Age Unknown	0.82	0.34	2.00
**Guniea 2012**	< 1 Year	0.88	0.52	1.47
	1-2 Years	0.74	0.53	1.03
	2-3 Years	0.91	0.66	1.26
	>3 Years	1.97	1.44	2.69
	Age Unknown	1.00	0.37	2.67
**Kenya 2015**	< 1 Year	0.64	0.51	0.81
	1-2 Years	0.77	0.61	0.99
	2-3 Years	0.75	0.54	1.05
	>3 Years	0.70	0.55	0.90
	Age Unknown	0.89	0.45	1.77
**Liberia 2009**	< 1 Year	0.90	0.75	1.09
	1-2 Years	0.83	0.56	1.22
	2-3 Years	0.86	0.52	1.41
	>3 Years	0.76	0.48	1.18
	Age Unknown	0.50	0.27	0.88
**Liberia 2011**	< 1 Year	0.97	0.78	1.21
	1-2 Years	1.01	0.79	1.30
	2-3 Years	1.07	0.70	1.64
	>3 Years	1.24	0.83	1.86
	Age Unknown	0.66	0.36	1.20
**Madagascar 2011**	< 1 Year	0.73	0.53	0.99
	1-2 Years	1.16	0.77	1.74
	2-3 Years	0.76	0.42	1.36
	>3 Years	0.79	0.48	1.28
	Age Unknown	1.36	0.62	2.86
**Madagascar 2013**	< 1 Year	1.05	0.74	1.49
	1-2 Years	0.82	0.50	1.32

	2-3 Years	1.04	0.67	1.60
	>3 Years	1.19	0.83	1.70
	Age Unknown	1.17	0.61	2.18
**Madagascar 2016**	< 1 Year	1.02	0.72	1.43
	1-2 Years	0.93	0.47	1.78
	2-3 Years	0.85	0.40	1.74
	>3 Years	0.62	0.29	1.29
	Age Unknown	0.98	0.43	2.12
**Mali 2012**	< 1 Year	0.93	0.76	1.13
	1-2 Years	1.13	0.92	1.39
	2-3 Years	1.06	0.78	1.44
	>3 Years	1.05	0.79	1.40
	Age Unknown	0.80	0.46	1.40
**Mali 2015**	< 1 Year	0.84	0.70	1.00
	1-2 Years	0.74	0.61	0.90
	2-3 Years	0.91	0.72	1.15
	>3 Years	1.00	0.82	1.22
	Age Unknown	1.04	0.50	2.14
**Mozambique 2011**	< 1 Year	0.96	0.77	1.19
	1-2 Years	0.93	0.72	1.20
	2-3 Years	0.96	0.65	1.42
	>3 Years	1.21	0.86	1.71
	Age Unknown	0.94	0.43	2.05
**Malawi 2012**	< 1 Year	0.92	0.65	1.29
	1-2 Years	0.85	0.61	1.19
	2-3 Years	0.65	0.45	0.94
	>3 Years	0.83	0.58	1.18
	Age Unknown	0.62	0.27	1.42
**Malawi 2014**	< 1 Year	0.77	0.56	1.07
	1-2 Years	0.76	0.55	1.04
	2-3 Years	0.57	0.40	0.83
	>3 Years	0.77	0.51	1.17
	Age Unknown	1.34	0.60	2.99
**Nigeria 2010**	< 1 Year	0.90	0.73	1.11
	1-2 Years	0.91	0.67	1.24
	2-3 Years	0.69	0.41	1.16
	>3 Years	1.28	0.86	1.92
	Age Unknown	1.24	0.55	2.79
**Nigeria 2015**	< 1 Year	1.15	0.96	1.38
	1-2 Years	0.84	0.67	1.06
	2-3 Years	1.13	0.80	1.60

	>3 Years	1.04	0.82	1.32
	Age Unknown	1.35	0.78	2.33
**Senegal 2011**	< 1 Year	0.83	0.53	1.29
	1-2 Years	1.09	0.64	1.80
	2-3 Years	0.82	0.37	1.75
	>3 Years	1.11	0.47	2.56
	Age Unknown	0.94	0.36	2.45
**Senegal 2012**	< 1 Year	0.57	0.32	0.99
	1-2 Years	0.71	0.45	1.09
	2-3 Years	0.56	0.35	0.87
	>3 Years	0.88	0.55	1.37
	Age Unknown	0.93	0.36	2.38
**Senegal 2014**	< 1 Year	1.21	0.70	2.07
	1-2 Years	1.01	0.51	1.95
	2-3 Years	0.97	0.45	2.00
	>3 Years	0.83	0.42	1.59
	Age Unknown	0.98	0.37	2.58
**Senegal 2015**	< 1 Year	0.65	0.34	1.18
	1-2 Years	1.23	0.68	2.20
	2-3 Years	0.70	0.32	1.46
	>3 Years	0.91	0.42	1.91
	Age Unknown	0.98	0.37	2.61
**Tanzania 2011**	< 1 Year	1.00	0.78	1.27
	1-2 Years	1.05	0.81	1.35
	2-3 Years	0.73	0.49	1.07
	>3 Years	0.84	0.55	1.26
	Age Unknown	1.21	0.80	1.82
**Tanzania 2015**	< 1 Year	1.07	0.88	1.31
	1-2 Years	0.59	0.39	0.88
	2-3 Years	1.17	0.76	1.77
	>3 Years	0.99	0.72	1.35
	Age Unknown	1.66	0.84	3.26
**Togo 2013**	< 1 Year	0.56	0.35	0.88
	1-2 Years	0.69	0.52	0.93
	2-3 Years	0.83	0.65	1.07
	>3 Years	0.87	0.68	1.11
	Age Unknown	0.86	0.38	1.90
**Uganda 2009**	< 1 Year	0.47	0.36	0.61
	1-2 Years	0.74	0.57	0.97
	2-3 Years	0.81	0.58	1.15
	>3 Years	0.64	0.45	0.91

	Age Unknown	0.66	0.36	1.20
**Uganda 2014**	< 1 Year	0.93	0.76	1.14
	1-2 Years	0.98	0.75	1.27
	2-3 Years	1.06	0.67	1.68
	>3 Years	0.67	0.42	1.08
	Age Unknown	1.02	0.73	1.43

Supplementary Table 5. Stratified results showing the effects of sleeping under an LLIN treated with different insecticides by survey.				
**Survey**	**Insecticide**	**Odds Ratio**	**Lower 95% UI**	**Upper 95% UI**
**Angola 2011**	Deltamethrin	0.80	0.51	1.22
	Unknown Insecticide	0.96	0.59	1.54
**Benin 2011**	Deltamethrin	0.73	0.55	0.95
	Permethrin	0.89	0.71	1.10
	Alphacypermethrin	0.78	0.47	1.29
	Unknown Insecticide	1.15	0.66	1.97
**Burkina Faso 2010**	Deltamethrin	1.04	0.88	1.22
	Permethrin	0.94	0.66	1.34
	Alphacypermethrin	0.82	0.64	1.05
	Unknown Insecticide	1.03	0.79	1.35
**Burkina Faso 2014**	Deltamethrin	0.88	0.72	1.07
	Permethrin	0.91	0.78	1.06
	Alphacypermethrin	0.72	0.49	1.04
	Unknown Insecticide	0.95	0.74	1.23
**Burundi 2012**	Deltamethrin	0.69	0.52	0.93
	Permethrin	0.62	0.32	1.18
	Alphacypermethrin	0.84	0.55	1.27
	Unknown Insecticide	0.94	0.36	2.43
**Cameroon 2011**	Deltamethrin	0.85	0.70	1.04
	Permethrin	1.06	0.75	1.48
	Alphacypermethrin	0.86	0.37	1.95
	Unknown Insecticide	0.66	0.44	0.99
**Cote d'Ivoire 2011**	Deltamethrin	0.70	0.56	0.88
	Permethrin	0.67	0.54	0.83
	Unknown Insecticide	0.74	0.47	1.18
**DR Congo 2013**	Deltamethrin	0.77	0.67	0.87
	Permethrin	1.05	0.82	1.36
	Alphacypermethrin	0.68	0.38	1.21
	Unknown Insecticide	1.18	0.69	2.01
**Gambia 2013**	Deltamethrin	0.58	0.34	0.97
	Unknown Insecticide	0.97	0.37	2.54
**Ghana 2014**	Deltamethrin	1.00	0.81	1.25
	Permethrin	0.85	0.52	1.39
	Alphacypermethrin	1.09	0.77	1.55
	Unknown Insecticide	1.35	0.60	3.02
**Guinea 2012**	Deltamethrin	1.22	0.92	1.61
	Permethrin	1.15	0.72	1.81

	Alphacypermethrin	1.12	0.82	1.53
	Unknown Insecticide	0.74	0.46	1.19
**Kenya 2015**	Deltamethrin	0.54	0.42	0.69
	Permethrin	0.74	0.62	0.90
	Unknown Insecticide	1.03	0.49	2.17
**Liberia 2009**	Deltamethrin	0.89	0.74	1.06
	Permethrin	0.67	0.48	0.94
	Unknown Insecticide	0.74	0.45	1.19
**Liberia 2011**	Deltamethrin	0.92	0.77	1.10
	Permethrin	1.08	0.59	1.99
	Alphacypermethrin	1.28	0.89	1.83
	Unknown Insecticide	1.53	0.79	2.97
**Madagascar 2011**	Deltamethrin	0.91	0.61	1.36
	Permethrin	0.72	0.51	1.00
	Alphacypermethrin	1.11	0.71	1.72
	Unknown Insecticide	0.78	0.49	1.21
**Madagascar 2013**	Deltamethrin	1.16	0.78	1.72
	Permethrin	1.05	0.75	1.45
	Alphacypermethrin	1.21	0.74	1.93
	Unknown Insecticide	0.91	0.61	1.34
**Madagascar 2016**	Deltamethrin	0.88	0.62	1.24
	Permethrin	1.12	0.61	2.02
	Alphacypermethrin	1.47	0.66	3.14
	Unknown Insecticide	0.63	0.31	1.24
**Mali 2012**	Deltamethrin	0.98	0.82	1.17
	Permethrin	1.09	0.88	1.35
	Alphacypermethrin	1.00	0.37	2.67
	Unknown Insecticide	1.01	0.64	1.58
**Mali 2015**	Deltamethrin	0.86	0.74	0.98
	Permethrin	0.70	0.42	1.15
	Alphacypermethrin	0.91	0.46	1.79
	Unknown Insecticide	0.96	0.68	1.35
**Mozambique 2011**	Deltamethrin	0.95	0.76	1.19
	Permethrin	1.04	0.84	1.28
	Unknown Insecticide	0.78	0.41	1.47
**Malawi 2012**	Deltamethrin	0.69	0.49	0.97
	Permethrin	0.93	0.67	1.29
	Alphacypermethrin	0.85	0.61	1.17
	Unknown Insecticide	0.54	0.26	1.12
**Malawi 2014**	Deltamethrin	0.78	0.49	1.25
	Permethrin	0.73	0.55	0.96

	Unknown Insecticide	0.88	0.38	1.99
**Nigeria 2010**	Deltamethrin	1.00	0.82	1.22
	Permethrin	0.92	0.70	1.22
	Alphacypermethrin	0.72	0.44	1.17
	Unknown Insecticide	0.81	0.47	1.37
**Nigeria 2015**	Deltamethrin	1.08	0.92	1.26
	Permethrin	1.10	0.80	1.52
	Alphacypermethrin	0.85	0.54	1.31
	Unknown Insecticide	1.05	0.82	1.34
**Senegal 2011**	Deltamethrin	0.87	0.58	1.30
	Permethrin	0.91	0.46	1.75
	Alphacypermethrin	1.16	0.48	2.74
	Unknown Insecticide	0.98	0.40	2.33
**Senegal 2012**	Deltamethrin	0.59	0.42	0.83
	Permethrin	0.65	0.38	1.11
	Alphacypermethrin	0.88	0.37	2.06
	Unknown Insecticide	1.07	0.49	2.25
**Senegal 2014**	Deltamethrin	0.73	0.40	1.30
	Permethrin	1.03	0.54	1.90
	Alphacypermethrin	1.05	0.41	2.63
	Unknown Insecticide	1.40	0.78	2.46
**Senegal 2015**	Deltamethrin	0.75	0.44	1.28
	Permethrin	0.71	0.34	1.42
	Alphacypermethrin	1.19	0.55	2.52
**Tanzania 2011**	Permethrin	1.00	0.81	1.24
	Alphacypermethrin	0.91	0.43	1.89
	Unknown Insecticide	0.70	0.38	1.27
**Tanzania 2015**	Deltamethrin	1.22	0.95	1.58
	Permethrin	1.05	0.86	1.28
	Alphacypermethrin	0.55	0.27	1.09
	Unknown Insecticide	0.56	0.31	0.97
**Togo 2013**	Deltamethrin	0.76	0.63	0.90
	Permethrin	0.83	0.38	1.78
	Unknown Insecticide	1.09	0.50	2.31
**Uganda 2009**	Deltamethrin	0.58	0.46	0.73
	Permethrin	0.66	0.50	0.88
	Alphacypermethrin	0.52	0.33	0.80
	Unknown Insecticide	1.04	0.60	1.81
**Uganda 2014**	Deltamethrin	0.86	0.69	1.07
	Permethrin	1.16	0.92	1.46
	Alphacypermethrin	0.99	0.60	1.62

Unknown Insecticide	0.63	0.43	0.93
